# Contamination of Drinking Water by Filtration of Organic Matter through the Soil†Read before the Sanitary Convention at Detroit, Jan. 7, 1880.

**Published:** 1880-06

**Authors:** Victor C. Vaughan

**Affiliations:** Lecturer on Medical Chemistry in the University of Michigan


### CONTAMINATION OF DRINKING WATER BY FILTRATION OF ORGANIC MATTER THROUGH THE SOIL.† By VICTOR C. VAUGHAN, M.D., PH.D., Lecturer on Medical Chemistry in the University of Michigan, assisted by P. E. NAGLE.

There seems to be a wide-spread belief that in disposing of decomposing organic matter it is only necessary to remove it from sight by burial in the earth. All through the rural districts and in the towns and many small cities of this country, privy-vaults, cess-pools and even cemeteries are located in close proximity to cisterns and wells. A few feet, or at most, a few rods of inter-

† Read before the Sanitary Convention at Detroit, Jan. 7, 1880.

vening soil are considered as sufficient to prevent the contamination of the water from these receptacles of decomposing matter. There is a general belief in the power of the soil to retain or to remove in some way all organic matter from solutions. Not infrequently a privy-vault may be seen located within ten or twenty feet of a well or cistern, and often the slope of the land is toward the well or cistern. During the past three months the authorities of a growing village in the interior of this State have, in spite of the remonstrance of certain citizens, located a cemetery within a few rods of a deep well, the water of which is used for household purposes.

During the summer of 1878, the sanitary condition of certain premises in Ann Arbor was as follows: The cistern, located at the rear of the house was about twenty feet deep, and about six feet below the surface it leaked. Of course if water could pass out, it could as readily pass in. Fifteen feet back in the yard, and slightly elevated, stood the privy. As soon as the warm months of summer came on, the water of the cistern became so offensive that it could not be used. The odor was that of the privy, and the water swarmed with microscopic animalculæ, and with others large enough to be seen with the unaided eye. All the water was removed and the cistern thoroughly cleansed. A few days later a heavy rain refilled the cistern, but within a few weeks more the water again became unfit for use, but was not so bad as it had been before the cleaning. During the remainder of the summer and fall the family obtained their drinking water from another source. During the winter season the water of the cistern so improved that after being filtered it could be used. But as soon as the warm weather of the following summer approached, the water again became unfit for use and was almost or quite as bad as it had been during the previous summer. This is only an illustrative case, and I know of similar ones in Ann Arbor.

Such instances as this caused us to doubt the power of soils of completely preventing the filtration of organic matter in solution. We determined to endeavor to answer, by experimental investigation, the following questions: (1.) To what extent are organic substances removed from solution by filtration through soil? (2.) Do different soils differ in their capability of thus removing organic matter? It must be noted here that the organic substances to be removed are held in solution, and not merely in suspension. The principal object in the filtration of natural waters for the supply of towns and cities is the removal of suspended matter. Again, the waters with which our experiments are concerned, and with which, as we shall endeavor to prove, drinking water is often contaminated, should be designated as *polluted* water, *i. e.*, the amount of injurious substances which they contain is sufficiently great to render their direct use dangerous.

The first thing necessary to an investigation of the first question, was to select some soluble organic substance, for which there are known exact methods of quantitative determination; for it would be necessary to determine the amount removed from solution by making quantitative determinations of the amounts in solution before and after filtration. For this purpose urea was selected, and, in order to have the conditions as natural as possible, the solution selected for filtration was urine. Urea is also easily decomposed, and experiments performed with this substance would have the advantage of the conditions being most favorable for the removal of the organic substance. It is well-known that urea is not far removed from inorganic matter and that its transformation into carbonic acid and ammonium is easily accomplished.

One cubic foot of the ordinary gravel soil, from four feet below the surface, was obtained and so arranged that fluids could be poured upon its surface (which was one foot square) and the filtrate collected. The amount of urea in a specimen of urine was then estimated with mercuric nitrate (the chlorides having previously been removed), a measured quantity of this specimen was poured upon the surface of the soil, and the urine which passed through was collected, measured and its contained urea estimated. A certain amount of urine (which was equal to that passed by one person in twenty-four hours) was poured upon the soil at one time. The filtrate from this was collected, measured and its contained urea estimated. This was repeated for a number of days or until the filtrate was found to contain as much urea per c. c., as the urine did before filtration. The results of these experiments are given in the following table:

| | Urea Poured on Soil. | Urine Passed Through | Total Urea Poured on in Solution. | Total Urea in the Filtrate. | Urea per 100 c. c. before Filtration. | Urea per 100 c. c. after Filtration. |
|---|---|---|---|---|---|---|
| | | | Grams. | Grams. | Grams. | Grams. |
| First Day... | 1,760 c. c. | 938 c. c. | 49.28 | 15.00 | 3.8 | 1.6 |
| Second Day. | 965 c. c. | 740 c. c. | 25.7 | 14.06 | 2.6 | 1.9 |
| Third Day .. | 860 c. c. | 600 c. c. | 13.76 | 9.60 | 1.6 | 1.6 |
| | 3,585 c. c. | 2,278 c. c. | 88.13 | 38.66 | | |

The soil, which had now become saturated with organic matter, was removed from the filter, spread out upon a clean board so as to be exposed to the air, left in this condition for twenty-four hours, and then returned to the filter. Urine was poured upon this soil, and the amount of urea removed by filtration was estimated as before. In this case it was found that the urine, before filtration, contained 2.1 grams of urea per 100 c. c., and after filtration, only 1.6 grams; or the soil which had been saturated with the organic matter was so far purified by exposure to the atmosphere for twenty-four hours that it now removed one half a gram of urea from every 2.1 grams poured upon it in solution.

From these experiments it is very evident that the ordinary gravel, which constitutes so large a proportion of the soil of this State, has but little effect in removing or oxidizing soluble nitrogenous substances from solutions which are allowed to pass through such soils. Furthermore, it became evident that such soil soon becomes saturated, and then no longer has any effect upon the removal or oxidation of these organic substances. The amount of urea necessary to thus saturate one cubic foot of soil was 88.13 grams, or that contained in 3,585 c. c. of urine. Of course this urine contained a small quantity of other organic substances, so we shall not claim that the following computations are exact; but we will be careful not to overstate them. Suppose that the total solid excretions of an adult are 112 grams per day (this of course is very low), then the solid excretions from a family of six persons each day would be sufficient, when properly dissolved, to saturate over seven cubic feet of gravel soil. From this it is evident that only a few weeks or months would suffice, with a proper amount of rain fall, to saturate every cubic foot of soil to the depth of five or ten feet in a small yard, in which we often find privy vault, cess-pool and cistern or well in close proximity. Of course if these substances are not destroyed by filtration, they will be carried with the water which passes through the soil wherever such water may go. If the water gains an entrance to the well it will carry with it the substances in solution. At this rate it would require more than a few feet or even rods of intervening soil to prevent the contamination of the water of wells or leaky cisterns from privy vaults, which often are not cleaned once a year, or from cess-pools or cemeteries.

But there is another consideration that must be mentioned in this connection. The gravel by itself forms one of the poorest of filters, because the particles are not all of the same size and shape, consequently the interspaces are also of unequal size, and the liquid, meeting with less resistance in one direction than in others, soon collects and forms streamlets which may convey the polluted water in quantity toward, or even into the source of the water used for household purposes.

This brings us to a consideration of the second question proposed to be investigated by experimental investigation, i. e., Do different soils differ in their capability of removing organic matter from solution? The gravel having been already tried we did not make any further experiments with it; but we next experimented with sand and loam in the same manner as we had done with the gravel. The comparative results only will be of interest. While urine (3,585 c. c.) containing 88.13 grams of urea, saturated one cubic foot of gravel, urine (4,000 c. c.) containing 89 grams of urea did not saturate one cubic foot of loam, but after this amount of urine had been used, the loam still removed four-tenths of a gram of urea from each 100 c. c. of urine. The sand was also better than the gravel but was not as good as the loam, for 88 grams of urea in 4,000 c. c of urine did not saturate the sand, which still removed two-tenths of a gram of urea from each 100 c. c. of urine. Thus of the three—gravel, sand and loam—the loam is most active, and the gravel least active in the removal of organic matter from solution.

The explanation of this may be conceived to be as follows: First, the inequality in size and variety in the shape of the pieces of gravel (as has already been referred to) allows the formation of streamlets, and the solution thus passes through the gravel more rapidly than through either the sand or loam. This is a physical defect in the gravel as an agent for the removal of the organic matter. A second cause is the greater porosity of the loam. As was shown by the renewed activity of the gravel after it had been exposed to the atmosphere for twenty-four hours, oxygen is the agent which acts chemically in the destruction of organic matter, or, rather, in its conversion into inorganic matter. Now the more concentrated the oxygen the more energetically will it act. As is well known, the atmosphere in the minute pores of the soil is condensed.

Oxygen has justly been termed the scavenger of creation, and the free access of this gas is necessary in order to render the excretions of animals harmless. This good work is begun in the living body; for the hæmoglobine of the red blood corpuscles carries oxygen to all the tissues, and by the action of this oxygen, poisons introduced from without and poisons generated within the organism are rendered inert and fitted for excretion.

Animals are formed by the agency of plants out of the constituents of earth and air. "Whatever in our bodies is inorganic originated in the soil; whatever in our bodies is organic originated in the atmosphere. Whatever in our bodies is inorganic will return to the soil; whatever in our bodies is organic will return to the atmosphere. The carbon of the carbonic acid of the air becomes the carbon of the cellulose, starch, gums, resins, etc., of plants; it becomes the carbon of our blood and muscle; and from our bodies it is returned to the air. Thus the changes of matter form an endless chain whose first link is also its last." (Gorup-Besanez.) Now the agent which converts the organic tissues of the animal into inorganic substances is oxygen; and this is the proper agent to use in rendering animal excretions harmless. This is the reason why the dry earth closet, with a pipe to conduct the gases into a chimney, is the best method (at least for the rural districts and small cities and villages) of disposing of fæcal matter. The dry earth takes up the oxygen, which is then condensed in the pores and acts with double energy. Instead of burying these substances down in the earth where but a limited supply of oxygen can reach them, it is far better to allow the free access of the atmosphere. Burial simply aids in the preservation of organic substances, and, as we have seen, they may be carried under the ground into our wells and thus poison us. Instead of simply burying these noxious substances and then thinking ourselves secure from them, it would be far better to render them inert and even useful, as may be done.

As to the location of cemeteries in the vicinity of wells, we think that our experiments show that the decomposing matter from one body would be sufficient to pass a long distance, especially through gravel soil, before it would be completely destroyed. We honor the dead as highly as others do, but it is not right that the dead should be allowed to murder the living.—*From the Sanitarian.*

---

PYROGALLIC ACID IN PHAGDENIC CHANCRE.—Vidal. (*Journ. de Méd.*, Feb., 1880, p. 68.)

The author has found a new use for pyrogallic acid, which he uses a good deal in treatment of skin diseases; that is, in the treatment of phagedenic chancres. In two cases the result was almost immediate. In the first case there was phagedenic chancre of rapid march, accompanied by a suppurating bubo, also phagedenic. The chancre was dressed with an ointment of pyrogallic acid and the bubo was touched only with the same; in a few days the ulceration was arrested. The second case was a chancre from inoculation and had become phagedenic, the size of a five franc piece. The same result was obtained in three days and cicatrization began. The ointment employed was at first 10 per cent., then 20 per cent. The strength should vary according to circumstances and the sensitiveness of the patient.

